# Ketamine exerts antidepressant effects and reduces IL-1β and IL-6 levels in rat prefrontal cortex and hippocampus

**DOI:** 10.3892/etm.2013.930

**Published:** 2013-01-29

**Authors:** CHUN YANG, TAO HONG, JIANG SHEN, JIE DING, XIONG-WEI DAI, ZHI-QIANG ZHOU, JIAN-JUN YANG

**Affiliations:** 1 Department of Anesthesiology, The Third Affiliated Hospital of Suzhou University, Changzhou;; 2 Department of Anesthesiology, Jinling Hospital, School of Medicine, Nanjing University, Nanjing, P.R. China

**Keywords:** ketamine, antidepressant effects, interleukin-1 β, interleukin-6, prefrontal cortex, hippocampus

## Abstract

Ketamine has fast-acting and robust antidepressant effects in animal models and depressed patients. It has been hypothesized that its underlying mechanism of action is associated with the inflammatory response in the central nervous system. Therefore, the present study was designed to investigate the antidepressant effects of ketamine and the expression of interleukin (IL)-1β and IL-6 in the prefrontal cortex and hippocampus of a rat model. Twenty Wistar rats were randomly divided into 2 groups (each group, n=10); the saline group and the ketamine group. On the 1st day, rats undertook a forced swimming test (FST) for 15 min (pre-test session). On the 2nd day, saline or ketamine was administered intraperitoneally 30 min before the test session. Following this, rats performed another FST for 5 min (test session) and the immobility time was recorded. The rats were then sacrificed, and the prefrontal cortex and hippocampus were harvested for determination of IL-1β and IL-6 levels. Compared with the saline group, ketamine administration significantly decreased the immobility time of rats during the FST (P<0.05). In addition, the ketamine group demonstrated a statistically significant decrease in the expression of IL-1β and IL-6 in rat prefrontal cortex and hippocampus compared with the saline group (P<0.05). Ketamine-induced antidepressant effects are associated with decreased levels of IL-1β and IL-6 in rat prefrontal cortex and hippocampus.

## Introduction

Depression is a debilitating disorder with severe health problems which pose a heavy economic burden to society (1). At present, monoamine drugs are the dominant therapeutic agent of choice for the treatment of depression (2). However, currently available antidepressant agents have severe drawbacks, such as low rates of treatment response and delayed onset time (3,4). In several studies, depression is considered as the disease that is related to the high expression of proinflammatory cytokines, and furthermore, attenuation of inflammation is suggested as one possible mechanism explaining the action of antidepressant agents (5–7). Collectively, the inflammatory response system is likely to be implicated in the etiology and treatment of depression.

Ketamine, an ionotropic glutamatergic N-methyl-D-aspartic acid (NMDA) receptor antagonist, is widely used in clinical anesthesia and pain treatment. Recent clinical studies have provided evidence that sub-anesthetic doses of ketamine may rapidly alleviate symptoms of depression, even for treatment-resistant depression, with an identical therapeutic effect (8,9). However, its underlying mechanisms have yet to be fully elucidated.

A previous review summarized that ketamine has the potential to modulate inflammation (10). It has been widely acknowledged that ketamine has been recommended for use in the surgery of sepsis patients due to its anti-inflammatory effects (11,12). However, too little attention is presently focused on the underlying mechanism with regard to whether inflammatory cytokines are involved in the antidepressant effects of ketamine.

Thus, we propose a hypothesis that ketamine may exert antidepressant effects via modulation of proinflammatory cytokines. Based on this theory, we aimed to validate the above-mentioned hypothesis. The present study was designed to determine the levels of IL-1β and IL-6 in the rat prefrontal cortex and hippocampus after ketamine administration during a forced swimming test (FST).

## Materials and methods

### Animals and drugs

Twenty male Wistar rats weighing 180–300 g were purchased from the Shanghai Animal Center (Shanghai, China). Five rats were housed per cage with food and water available *ad libitum* and maintained on a 12-h light/dark cycle (lights on at 07:00 am). Rats were randomly divided into 2 groups (each group, n=10). Rats were intraperitoneally administered with saline or ketamine at a dose of 10 mg/kg 30 min before the test session of FST. Ketamine was obtained from the Gutian Pharmaceutical Company (Fujian, China). The experimental procedures were approved by the Institutional Animal Ethics Committee of Suzhou University (Suzhou, China).

### FST

FST was applied according to our previous study (13) to evaluate the antidepressant effects of ketamine. The test included two separate sessions in a cylindrical tank (30-cm diameter, 60-cm height) filled with water (22–23°C) to a 30-cm level in which rats were unable to touch the bottom of the tank. The water was replaced after each rat had completed one session. All procedures were conducted between 9:00 am–15:00 pm. Rats first underwent a FST for 15 min (pre-test session). After 24 h, rats were placed in the water again for 5 min (test session), and the immobility time was recorded in seconds. Immobility was defined as the amount of time that the rat remained floating in the water without struggling and made only those movements necessary to keep its head above the water.

### Testing IL-1β levels

Following FST, rats were immediately sacrificed and the prefrontal cortex and hippocampus were harvested for determination. In brief, the prefrontal cortex and hippocampus were separately washed with ice-cold phosphate-buffered solution (PBS) and scraped in lysis buffer. The insoluble material was removed by centrifugation at 12,000 rpm for 20 min. Protein content was measured according to the bicinchoninic acid (BCA) method. Non-specific binding was blocked for 1 h at 37°C in TBS containing non-fat dried milk. The membrane was then incubated with primary antibodies against IL-1β (1:1000) and β-actin served as the control. Membranes were then incubated in the appropriate HRP-conjugated secondary antibodies (1:20000).

### Testing IL-6 levels

IL-6 levels in the prefrontal cortex and hippocampus were measured by sandwich-ELISA of relevant primary antibodies according to the manufacturer’s instructions (Chemicon, Temecula, CA, USA). Briefly, rat prefrontal cortex and hippocampus were homogenized in PBS with 1 mM phenylmethylsulfonyl fluoride (PMSF) and 1 mM ethylene glycol tetraacetic acid (EGTA). Microtiter plates (48-well flat-bottom) were coated for 24 h with the samples diluted 1:2 in sample diluent and standard curves ranged between 7.8 and 500 pg/ml. The plates were then washed four times with sample diluent and a monoclonal rabbit antibody diluted 1:1000 in sample diluent was added to each well. The plates were then incubated for 3 h at room temperature. After washing, a peroxidase-conjugated anti-rabbit antibody (diluted 1:1000) was added to each well and incubated at room temperature for 1 h. After addition of streptavidin-enzyme, substrate and stop solution, the amount of IL-6 was determined by absorbance at 450 nm. The standard curve demonstrates a direct correlation between optical density (OD) and the concentration of IL-6. Total protein was measured by Lowry’s method using bovine serum albumin as a standard.

### Statistical analysis

Data are presented as mean ± standard deviation (SD). Statistical analyses were performed by one-way analysis of variance (ANOVA). These statistical analyses were conducted by Statistical Product for Social Sciences (SPSS version 17.0). P<0.05 was considered to indicate a statistically significant result.

## Results

### Effects of ketamine on the immobility time of rats during FST

**Figure 1:**
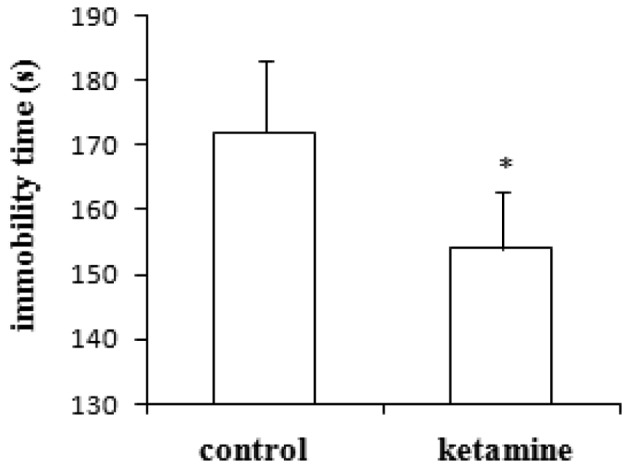
Effects of ketamine on the immobility time (mean ± SD) of rats during FST. Each group contained ten subjects. ^*^P<0.05, compared with the saline group. FST, forced swimming test.

Compared with saline administration, the administration of ketamine significantly decreased the immobility time of rats during FST (P<0.05; Fig. 1).

### Effects of ketamine on the expression of IL-1β in rat prefrontal cortex and hippocampus

**Figure 2:**
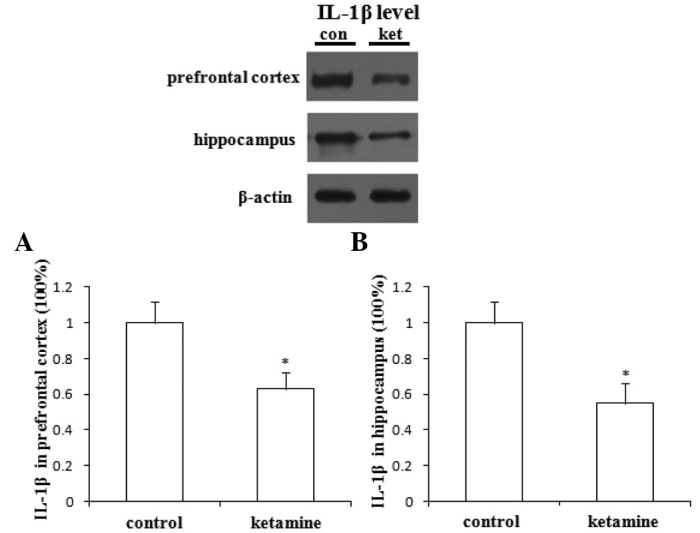
Effects of ketamine on the expression of IL-1β in rat (A) prefrontal cortex and (B) hippocampus. Each group contained ten subjects. ^*^P<0.05, compared with the saline group. IL, interleukin.

Compared with saline administration, the administration of ketamine significantly decreased the expression of IL-1β in rat prefrontal cortex (Fig. 2A) and hippocampus (Fig. 2B; P<0.05).

### Effects of ketamine on the expression of IL-6 in rat prefrontal cortex and hippocampus

**Figure 3:**
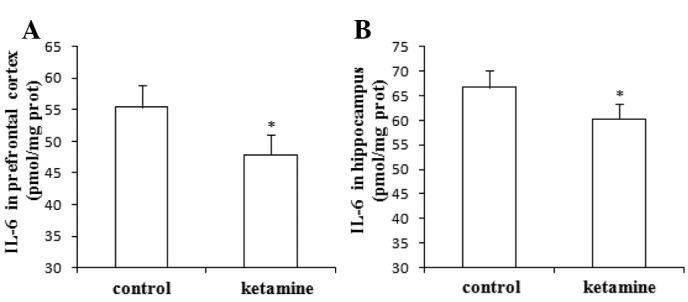
Effects of ketamine on the expression of IL-6 in rat (A) prefrontal cortex and (B) hippocampus. Each group contained ten subjects. ^*^P<0.05, compared with the saline group. IL, interleukin.

Compared with saline administration, the administration of ketamine significantly decreased the expression of IL-6 in rat prefrontal cortex (Fig. 3A) and hippocampus (Fig. 3B; P<0.05).

## Discussion

In the present study, we demonstrated that ketamine significantly decreased the immobility time of rats in FST, and there was a significantly lower expression of IL-1β and IL-6 in rat prefrontal cortex and hippocampus following ketamine administration.

Recent animal and clinical studies have indicated that the administration of ketamine at sub-anesthetic doses has fast-acting and robust antidepressant effects (8,9), and it has been shown that ketamine, widely used as an anesthetic agent, demonstrates unique effects for the treatment of depression. In the present study, the administration of ketamine at a dose of 10 mg/kg significantly decreased the immobility time of rats receiving FST. This result is consistent with previous studies and confirmed ketamine’s antidepressant effects.

It has previously been reported that ketamine is recommended for use in the anesthesia of patients with sepsis due to its effects of stimulating sympathetic nerves, maintaining vasoconstriction and maintaining circulation stability (14,15). Taniguchi and Yamamoto (16) have indicated that ketamine may inhibit the sepsis-induced inflammatory response in a rat model and attenuate the fall of blood pressure. Furthermore, a previous study conducted by Sun *et al* (17) has revealed that ketamine’s anti-inflammatory effects may be associated with the changes in the expression of nuclear factor-κB and tumor necrosis factor-α. Collectively, these findings suggest that ketamine has significant anti-inflammatory effects.

At present, the treatment of depression is mainly dependent on conventional antidepressant agents. A large body of evidence suggests that the peripheral serum proinflammatory cytokine levels of patients with depression are significantly higher than normal levels, and the increased levels are proportional to the severity of depression (18,19). In addition, it has been demonstrated that the proinflammatory cytokine levels in the peripheral blood of patients with depression gradually tend to become normal following treatment with antidepressant agents (20,21). In the preliminary study, we observed a lower expression of IL-1β and IL-6 in rat prefrontal cortex and hippocampus after ketamine administration, and the results validated our above-mentioned hypothesis.

Mounting studies reveal that both IL-1β and IL-6 are important in the etiology and pathophysiology of depression (22,23). In clinical trials, elevated levels of IL-1β and IL-6 have been observed in the peripheral blood of depressed patients (23,24). Besides, in experimental animals, administration of IL-1β has produced depressive-like symptoms which were attenuated by treatment with antidepressants (25,26). Therefore, we concluded that the onset of antidepressants may be accompanied by the inhibition of IL-1β and IL-6. In the present study, the results demonstrated that ketamine exerts an antidepressant effect that is associated with the downregulation of IL-1β and Il-6 levels, which was consistent with the above-mentioned conclusion.

In conclusion, ketamine possesses properties that exert antidepressant effects, which have been confirmed in the present study, and its underlying mechanism is potentially associated with the inhibition of IL-1β and IL-6 expression in rat prefrontal cortex and hippocampus. However, a great limitation of the present study is that we did not test the levels of anti-inflammatory cytokines, which future studies are required to investigate further.
